# Identification of a Common Gene Expression Response in Different Lung Inflammatory Diseases in Rodents and Macaques

**DOI:** 10.1371/journal.pone.0002596

**Published:** 2008-07-09

**Authors:** Jeroen L. A. Pennings, Tjeerd G. Kimman, Riny Janssen

**Affiliations:** 1 Laboratory for Health Protection Research (GBO), National Institute for Public Health and the Environment (RIVM), Bilthoven, The Netherlands; 2 Central Veterinary Institute, Lelystad, The Netherlands; The Rockefeller University, United States of America

## Abstract

To identify gene expression responses common to multiple pulmonary diseases we collected microarray data for acute lung inflammation models from 12 studies and used these in a meta-analysis. The data used include exposures to air pollutants; bacterial, viral, and parasitic infections; and allergic asthma models. Hierarchical clustering revealed a cluster of 383 up-regulated genes with a common response. This cluster contained five subsets, each characterized by more specific functions such as inflammatory response, interferon-induced genes, immune signaling, or cell proliferation. Of these subsets, the inflammatory response was common to all models, interferon-induced responses were more pronounced in bacterial and viral models, and a cell division response was more prominent in parasitic and allergic models. A common cluster containing 157 moderately down-regulated genes was associated with the effects of tissue damage. Responses to influenza in macaques were weaker than in mice, reflecting differences in the degree of lung inflammation and/or virus replication. The existence of a common cluster shows that *in vivo* lung inflammation in response to various pathogens or exposures proceeds through shared molecular mechanisms.

## Introduction

Microarray gene expression profiling has become a common method for gaining insight into molecular disease mechanisms that are involved in host-pathogen interaction, and the outcome of the infection process, in terms of development of disease. The increasing public availability of microarray data allows for combining data in a meta-analysis, to identify common clusters of genes induced upon infection. Since most innate immune responses, especially those to pathogen-associated molecular patterns, are evolutionary conserved, it is likely that such common responses can be found. Indeed, it has been shown that under controlled *in vitro* conditions macrophages respond to a broad range of bacteria with a shared gene expression pattern [1] and similar findings have been described for dendritic cells [2] and peripheral blood mononuclear cells [3]. The meta-analysis of *in vitro* data by Jenner and Young [4] revealed a common infection cluster across a larger number of pathogens and studies, that included several genes for which this role was not recognized in the underlying studies. However, whether these findings in *in vitro* infection models are representative for what happens *in vivo* is unknown. Our laboratory recently published data on *in vivo* lung infection responses to respiratory syncytial virus (RSV) [5] and *Bordetella pertussis* [6], [7]. Comparing these data sets showed several similarities as well as differences in the genes involved, although the kinetics of the responses was completely different [5]–[7]. To make additional comparisons and identify a common set of upregulated genes in different inflammatory diseases of the lung, we collected additional data for acute lung inflammation models from literature studies and other studies at our institute. As interpreting pair wise comparison between models is hampered by the large data size for each study, our goal was to use the data in a meta-analysis. Because the number of different pathogens or other exposures in each group is small compared to *e.g.* toxicogenomics experiments, it is not possible to determine pathogen-specific responses with sufficient certainty. The dataset is, however, suitable to detect responses common to all – or to a substantial number of – pathogens, or to reflect the “acute phase response” in the lung. Therefore, our aim was to employ a meta-analysis to identify gene expression changes in *in vivo* lung inflammatory models that are common to all, or subsets of, inducers of lung inflammatory lesions and pathogens.

## Results

We combined microarray data from 12 studies [5]–[17] to compile a table containing gene expression data for 4551 genes upon 45 treatment conditions causing pulmonary pathology. These include 4 exposures to chemical (i.e. air pollution) sources, namely ozone and particulate matter (PM); 12 to bacterial infection models (including LPS, a mimic of infection); 19 to viral infections; 5 to parasitic infections; and 5 to allergic asthma models. More details on these exposures are given in Table 1. Hierarchical clustering on the data showed that there was one cluster showing a similar response pattern across the rodent and macaque pulmonary inflammation models. This cluster contains 383 genes and is available as supplementary data at the journal's website (Supporting Dataset S1). Please note that, because gene symbols for rodents and macaques differ in which typecase is used, we will refer to genes in uppercase throughout this paper and supplementary data to connect to conventions for writing human gene symbols.

**Table 1:** Data and studies used in the meta-analysis; including labels used in Figure 1.

Source data	Species	Exposure	Time points, labels
*Chemical*			
Kooter, 2007	mouse	ozone	A: 12 h
Kooter, 2005	rat	Particulate matter	B: 2–6 h, C: 15–21 h, D: 24–40 h
*Bacterial*			
Banus, 2007	mouse (two strains : C3H and HcB28)	*Bordetella pertussis*	A: C3H 1 d, B: C3H 3 d, C: C3H 5 d, D: HcB28 1 d, E: HcB28 3 d, F: HcB28 5 d
Lewis, 2008	mouse	LPS aerosol	G: 4 h
		*Mycoplasma pulmonis*	H: 7 d
		*Pseudomonas aeruginosa*	I: 2 h
Rosseau, 2007	mouse	*Streptococcus pneumoniae*	J: 1 d, K: 2 d, L: 4 d
*Viral*			
Janssen, 2007	mouse	Respiratory Syncytial Virus	A: 1 d, B: 3 d
Kash, 2004	mouse	Influenza (three strains: 1918, NC, and WSN)	C: 1918 1 d, D: 1918 3 d, E: NC 1 d, F: NC 3 d, G: WSN 1 d, H: WSN 3 d
Rosseau, 2007	mouse	Influenza	I: 1 d, J: 2 d, K: 4 d
Baskin, 2004	macaque	Influenza	A: 4 d, B: 7 d
Kobasa, 2007	macaque	Influenza (two strains: K173 and 1918)	C: K173 3 d, D: K173 6 d, E: K173 8 d, F: 1918 3 d, G: 1918 6 d, H: 1918 8 d
*Parasitic*			
Lewis, 2008	mouse	*Nippostrongylus brasiliensis*	A: 5 d
Reece, 2006	mouse	*Nippostrongylus brasiliensis*	B: 2 d, C: 3 d, D: 4 d, E: 8 d
*Allergic asthma*			
Kuperman, 2005	mouse	OVA	A: 1 d
Lewis, 2008	mouse	OVA	B: 1 d
		*Aspergillus* extract	C: 4 h
Zimmermann, 2003	mouse	OVA	D: 18 h
		*Aspergillus* extract	E: 18 h

Initial inspection showed that among the 383 genes there were a considerable number of immune and inflammation-related genes. Gene Ontology (GO) term enrichment analysis by the DAVID resource website (http://david.abcc.ncifcrf.gov/) [18], [19] showed that 100 of the 383 genes are annotated with the GO-term “immune response” (Fisher Exact p-value = 4.1E-43) and 40 genes are annotated with the term “inflammatory response” (Fisher Exact p-value = 1.1E-29) (Supporting Dataset S1). Also, the list shows substantial overlap with genes previously identified in *in vitro* experiments on host-pathogen responses. This includes 120 genes that were previously found by Jenner and Young [4] as being involved in the host-pathogen response; 82 genes described by Huang *et al.* [2] as dendritic cell common responsive genes; 42 genes described by Nau *et al.* [1] as being induced as part of the macrophage activation program; and 30 genes described by Boldrick *et al.* [3] as common responsive genes in peripheral blood mononuclear cells (Supporting Dataset S1). The largest overlap between the *in vivo* and *in vitro* responses is found for inflammatory cytokines and chemokines.

Within the common up-regulated set of 383 genes, several immunological processes are represented by a substantial number of genes. These can be summarized in order of decreasing overlap with the *in vitro* studies.

The functional class of genes that is most prominently up-regulated consists of cytokines and chemokines. Among these genes are interleukins (*IL1A*, *IL1B*, *IL5*, *IL6*, *IL12B*, *IL13*, *IL15*), CC-chemokines (*CCL2* (*MCP1*), *CCL4* (*MIP-1B*), *CCL5* (*RANTES*), *CCL7* (*MCP3*), *CCL8* (*MCP2*), *CCL11* (eotaxin), *CCL17* (*TARC*), *CCL19* (*MIP-3B*), *CCL20* (*MIP-3A*), *CCL22*), CXC-chemokines (*CXCL1* (*GRO1*), *CXCL2* (*MIP-2A*), *CXCL5*, *CXCL9* (*MIG*), *CXCL10* (*IP-10*), *CXCL13*) and other cytokines such as *CSF1* (*M-CSF*), *CSF3* (*G-CSF*), *TNF*, *SPP1* (osteopontin), *AREG* (amphiregulin). These genes not only show a consistent up-regulation in *in vivo* pulmonary inflammation studies, but also a clear overlap with genes found to be induced *in vitro* [1]–[4] (Supporting Dataset S1), corroborating the pivotal role these genes play in response to a wide range of agents.

A second major group among the up-regulated genes are interferon-induced genes. These include guanylate binding proteins 1 and 2 (*GBP1* and *GBP2*), myxovirus resistance 1 and 2 (*MX1* and *MX2*), chemokines *CXCL9* and *CXCL10*, indoleamine-pyrrole 2,3 dioxygenase (*INDO*), and tryptophanyl-tRNA synthetase (*WARS*). In addition, these are several functionally less well characterized interferon-induced genes such as *IFI27*, *IFI30*, *IFI35*, *IFIT1*, *IFIT2*, and *IFITM3*. This set of genes contains both IFNα/β and IFNγ regulated genes. Other markers that are consistent with activation of the IFNγ response are several immunoproteasome components (*PSMB8*, *PSMB9*, *PSMB10*, *PSME2*). Increased expression of interferon-induced genes is mainly found for bacterial and viral infection models, illustrating the role of interferon in the innate immunity system's first line of defense against both viral and bacterial pathogens. Most of the genes in this class are also induced in *in vitro* models (Supporting Dataset S1).

Although not as strongly induced as the previous classes, several genes involved in immunological signaling pathways were found to be consistently up-regulated. These belong to pathways such as interferon signaling (*IFNAR2*, *IRF1*, *IRF4*, *IRF7*, *ISGF3G*, *JAK2*, *STAT1*, *STAT2*), NF-κB signaling (*NFKB2*, *NFKBIB*, *NFKBIE*, *IKBKE*, *REL*, *RELB*, *TNFAIP3*), AP-1 signaling (*JUNB*, *FOS*, *FOSL1*, *FOSL2*), MAPK signaling (*MAPK13*, *MYC*), and TLR signaling (*TLR2*, *CD14*, *MYD88*, *PIK3CD*, *PIK3CG*, and *TBK1*). These pathways are interconnected, and several of the genes mentioned play a role in more than one signaling process. These interconnections between the various signaling routes makes the immunological signaling as a whole more robust and may also explain why the common *in vivo* response extends across all these pathways and even several dozens of other transcription factors that are not directly connected to these pathways. It is interesting to note that although the majority of the common up-regulated signaling genes activate the immune response, several inhibitory genes are also found for the NF-κB signaling pathway, namely *NFKBIB*, *NFKBIE*, *TNFAIP3*. The expression patterns for these three genes are similar to other signal transduction genes. This indicates that the induction of these inhibitory genes is an equally important aspect of the response, as these genes will help restore the host cell to its normal state when the inflammatory stimulus is no longer present, thus keeping the system in check. For the immunological signaling genes, the overlap with *in vitro* studies is less pronounced than for cytokines and chemokines and interferon-induced genes.

In addition to these three groups of immune-related genes, there are several smaller gene classes involved in the immune or infection response. These include known inflammation markers (such as *S100A8*, *S100A9*, *LCN2*, *SAA2*), genes involved in the complement cascade (*C1QA*, *C1QB*, *C3*, *C3AR1*, *C5R1*), cytotoxicity (*GZMA*, *GZMB*), or tissue remodeling (*CHI3L1*, *MMP8*, *MMP9*, *MMP12*, *MMP15*). Others play a role in immune cell adhesion such as *VCAM1*, integrins (*ITGAM*, *ITGAX*, *ITGB2*), or selectins (*SELE*, *SELL*, *SELP*). Finally, some genes are not exclusive to the immune system but connect this to other cellular functions such as genes involved in leukotriene and prostaglandin metabolism (*ALOX5AP*, *ALOX12*, *ALOX15*, *PTGS2*, *PTGES*), nitric oxide metabolism (*NOS2* (*iNOS*) and *ARG2*), or protection against oxidative stress (*SOD2*, *HMOX1*). Taken together, these processes play various roles in the immune or inflammatory response. The finding that a broad range of immune-related processes are induced across the different exposure categories shows that the *in vivo* pulmonary inflammatory response to various pathogens or exposures proceeds through – at least partly – shared molecular mechanisms.

Apart from genes involved in immune or infection-related processes, we also found a substantial number of genes involved in cell division or proliferation. These include *CCNA2*, *CCNB1*, *CCNF*, *CDC2A*, *CDC6*, *CDC20*, *CDKN1A* (*P21*), *CDKN2D*, *AURKB*, *BUB1B*, *MKI67*, and *UBE2C*. For these genes, the strongest induction was observed for parasitic (helminth) and protein sensitization (allergic asthma) models. Surprisingly, there was practically no overlap between these genes and the genes mentioned in *in vitro* studies, suggesting a mechanism specific for the *in vivo* response.

**Figure 1:**
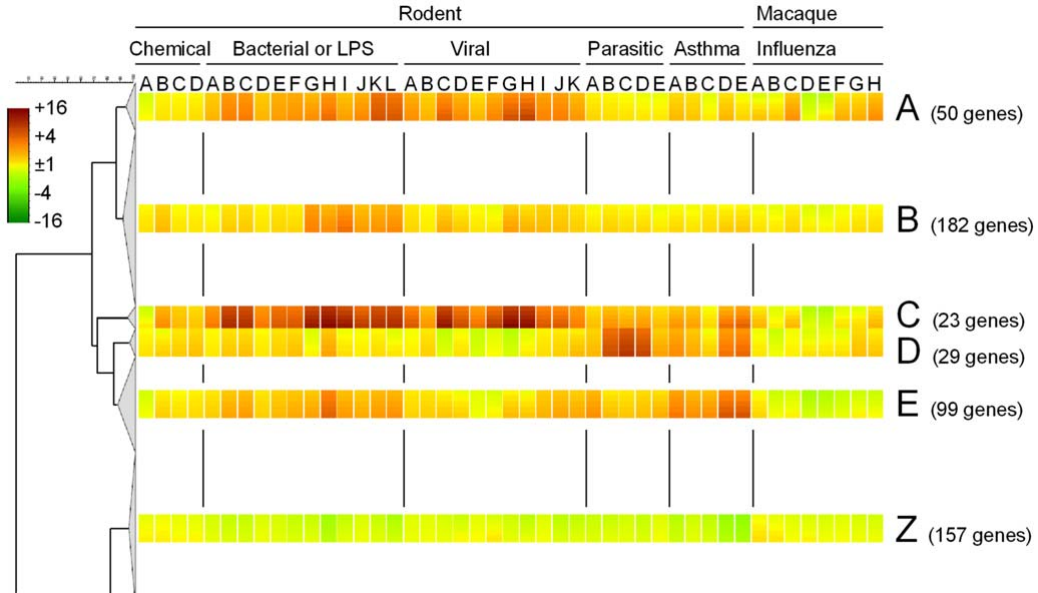
Gene sets with common expression responses. Fragment of the hierarchical clustering dendrogram containing the abridged clusters for five common up- (A–E) and one down-regulated (Z) gene cluster. Expression changes compared to control levels are indicated by the color bar and corresponding ratio: green represents down-regulation, yellow no difference, red up-regulation. The heat map color gradient within each block indicates the variation within that block. Full details on the exposures used and the corresponding labels can be found in Table 1 and Supporting Table S1.

To further characterize gene expression response patterns, the full 4551-gene dendrogram was reduced to the most significant branches. Using the GeneMaths option Cluster Plot (see Methods section for details) provided a recommended branch cutoff level of 88% cluster similarity. Clipping the dendrogram at this level resulted in a total number of fifteen branches, ranging in size from 23 to 1713 genes. Most of these show no apparent induction or down-regulation upon exposure or show only a (moderate) change within a single study (data not shown). Five branches – which together form the common pulmonary inflammation response cluster of 383 genes – show an induction pattern that is consistent across multiple studies and/or exposures (Fig. 1, subset A–E). These five subsets display different levels and/or patterns of induction (Fig. 1, subset A–E).

Among the five subsets group C, containing 23 genes, shows the strongest and most common response to lung inflammation in rodents, even extending to particulate matter. In primate influenza models this subset is also up-regulated, although not as strong as in rodent models. GO-term enrichment showed that this is the only subset clearly enriched for inflammatory response genes (Supporting Dataset S1). This is also reflected in the regulated genes (Table 2), which include several known genes involved in inflammatory response such as the TLR4 co-receptor *CD14*; inflammatory cytokines such as *IL1B* and *IL6*; and chemokines like *CCL2*, *CCL4*, *CXCL9*, and *CXCL10*. Additionally, several well-known inflammation markers were present, including *S100A8* and *S100A9* (Calgranulin A and B), *LCN2* (lipocalin 2 or *NGAL*), and serum amyloid A2 (*SAA2*). Pathway analysis by MetaCore (GeneGo, San Diego, CA) showed a high rank for the pathway “Toll-like receptor (TLR) ligands and common TLR signaling pathway leading to cell proinflammatory response”, which is consistent with up-regulation of proinflammatory cytokines and chemokines.

**Table 2:** Subset C of the common lung inflammation response, containing the 23 genes with the strongest and most common response across the various pathogens and exposures.

Symbol	Gene Name
CCL2	Chemokine (C-C motif) ligand 2
CCL4	Chemokine (C-C motif) ligand 4
CCL7	Chemokine (C-C motif) ligand 7
CD14	CD14 antigen
CXCL1	Chemokine (C-X-C motif) ligand 1
CXCL2	Chemokine (C-X-C motif) ligand 2
CXCL5	Chemokine (C-X-C motif) ligand 5
CXCL9	Chemokine (C-X-C motif) ligand 9
CXCL10	Chemokine (C-X-C motif) ligand 10
FCER1G	Fc receptor, IgE, high affinity I, gamma polypeptide
FCGR3	Fc receptor, IgG, low affinity III
GBP2	Guanylate binding protein 2, interferon-inducible
IFIT1	Interferon-induced protein with tetratricopeptide repeats 1
IL1B	Interleukin 1, beta
IL1RN	Interleukin 1 receptor antagonist
IL6	Interleukin 6 (interferon, beta 2)
LCN2	Lipocalin 2
S100A8	S100 calcium binding protein A8 (calgranulin A)
S100A9	S100 calcium binding protein A9 (calgranulin B)
SAA2	Serum amyloid A2
TGFBI	Transforming Growth Factor, beta-Induced, 68 kDa
TGTP	T-cell specific GTPase
TIMP1	TIMP metallopeptidase inhibitor 1

A second subset showing a general response to pulmonary inflammation is group A. This subset is particularly induced upon bacteriological and viral infections in mice. Functional and pathway analysis showed that the genes in this subset are especially involved in interferon signaling, with the three highest-ranking MetaCore pathways being “Antiviral actions of Interferons”, “IFN alpha/beta signaling pathway”, and “Antigen presentation by MHC class I”. The last of these pathways is also mediated by interferon gamma through the formation of immunoproteasomes and the synthesis of the proteasome activator PA28 [20]. Among the 50 genes in this cluster are genes involved in interferon signaling such as *STAT1* and *STAT2*, and more than 15 interferon-induced proteins such as myxovirus resistance 1 (*MX1*), tryptophanyl-tRNA synthetase (*WARS*), and indoleamine-pyrrole 2,3 dioxygenase (*INDO*) (Supporting Dataset S1).

Subset D, containing 29 genes, shows mainly a gene expression response in parasitic and asthma models in mice. Both of these models are associated with T helper 2 (Th2) responses [15], [17]. DAVID and MetaCore revealed that this is the only subset that is not enriched for immunological genes. Instead, it is enriched for cell cycle related genes, especially those involved in cell division such as cyclins A2, B1, F, and antigen *MKI67*.

Finally, there are two subsets with a general, although less pronounced, response. The larger of these is subset B, containing 182 genes. MetaCore indicated that this subset is enriched for several cytokine and chemokine signaling pathways, such as NF-AT, NF-κB, and MAPK signaling. This is reflected by the presence of genes such as *IL5*, *IL13*, *NFKB2*, *NFKBIB*, and *MAPK13* in this cluster.

The smaller of the two subsets is cluster E, containing 99 genes. DAVID and MetaCore analysis indicated that this subset contains a broad range of immunological genes. High ranking MetaCore pathways were “Classic complement pathway”, “Lectin Induced complement pathway”, and “Alternative complement pathway”, based on complement and integrin genes such as *C1QA*, *C1QB*, *C3*, *C3AR1*, *ITGAM*, *ITGAX*, and *ITGB2*. Other immunological genes in this subset include *CCL8*, *AREG*, and *NFKBIE*. Unlike the other subsets, which are induced in rodents and (albeit to a lesser extent) in macaques, this subset is not induced in macaques but only in the rodent models.

In addition to the common set of up-regulated genes, we also identified a cluster that displayed a general down-regulation. This cluster (which we will refer to as subset Z) contained 157 genes. DAVID and MetaCore showed this subset was enriched for development-related terms such as cell differentiation, organ development, blood vessel development, and growth factor activity. Some of the genes involved in these processes include *BDNF*, *BMP4*, *FGF1*, *FIGF*, *IGFBP3*, *TNNI3*, and *WNT3A*. Other processes that were overrepresented in this subset are muscle contraction and several metabolic processes. Only three immunological genes (*CD81*, *PLUNC*, *EFNB1*) were found among this subset. A complete listing of this subset is available as supplementary data at the journal's website (Supporting Dataset S2).

## Discussion

Combining gene expression data from multiple studies creates the possibility to compare effects and look for common or specific responses. In this study, we focused on *in vivo* acute lung inflammation models. We included allergic asthma models and exposures to air pollutants, as these also cause pulmonary inflammation and therefore provide gene expression data to which the nature and the extent of infection responses can be compared. When data from different studies are combined, it should be kept in mind that not al studies are equally comparable, as there are differences between inflammation models as well as between species, time points, as well as other practical details on how the study was performed. However, combining studies also results in a larger data set, which allows for an analysis to reveal additional information that would not be apparent in the original studies used. When more microarray data on pulmonary inflammation models will become available in the future, it can therefore be expected that the number of identifiable common and specific responsive genes and pathways will increase.

When different studies employ different methods in analyzing raw data this can cause unwanted (study-dependent) differences on the normalized data. For this reason we used the same normalization procedure on all raw two-color array data. Downloaded Affymetrix data were already normalized according to standard methods. Also, as the included studies used several kinds of microarrays, the initially collected data contain a large number of genes (mostly ESTs) for which only data from one or two studies are available. Therefore, to reduce the influence of missing data on the analysis, we also applied a filtering on the set of included genes, as described in the Methods section. The criteria were chosen so that a sufficiently large number of genes was included and small adjustments to the criteria had only a minor effect on the resulting clustering (data not shown).

The data used contained information for 45 compared exposures that could be grouped into five main categories, namely chemical (air pollution related), bacterial, viral, parasitic, and allergic asthma models. These data led us to identify a common cluster of 383 genes with a similar *in vivo* response pattern characterizing acute lung inflammation. Of these 383 genes, 120 were previously identified as belonging to an *in vitro* common infection response [4]. Within this cluster there were subsets associated with more specific functional roles such as the response to bacterial and viral infection (subset A), cytokine and chemokine signaling (subset B), general inflammatory response (subset C), and the response to parasites and allergic asthma models (subset D). A closer analysis on these subsets could enable us to identify new genes that are of mechanistic importance and suitable as biomarkers to evaluate infection with unknown pathogens.

When we compare gene expression profiles for the various exposures, it becomes apparent that the differences within each treatment category were smaller than those between categories. This indicates that in addition to a general common infection response there are additional, more dedicated, responses for categories of pathogens or treatments. An example of this is the difference we observed between responses to parasitic versus bacterial or viral infection. Although further elucidation of such responses would require data from a larger number of infection studies, the five subsets identified could serve as an initial starting point to see which processes are associated with a shared infection response to all or some categories of exposure.

Among the chemical exposures, the most pronounced response was observed for subset C. This subset is involved in a general inflammatory response to pathogens, allergic asthma, and even air pollutants. An inflammation-related gene expression response to air pollutants corresponds to the finding that both particulate matter (PM) and ozone cause lung inflammatory and cytokine responses [11], [12]. The response to ozone in the several subsets is both up- and down-regulated. This can be explained by the finding that in addition to an inflammatory response, ozone also causes suppression of immune responsive genes [12], [21]. It has been suggested that the inflammatory response to PM is caused by the presence of bacterial endotoxins such as LPS in particulate matter [22]–[24]. Indeed, the response to PM correlates best with the bacterial infection models (including a mimic of infection, LPS), as is visualized in Fig. 1. Our data underline that the response to LPS may be an important element of the response to PM.

Bacteria induce gene expression in several subsets, and the response to LPS aerosol matches the other responses to bacterial infection in these subsets. Even though LPS aerosol is not an actual infection, it mimics exposure to a pathogen and accordingly induces an inflammatory response. Interestingly, this response is not strictly LPS-specific as the bacterial infections used include not only gram negative (*Bordetella*, *Pseudomonas*) bacteria, but also the LPS-lacking *Mycoplasma* and *Streptococcus*. This indicates that the response to bacterial infection is not only dependent on LPS signaling via TLR4 but signaling through other Toll-like receptors also plays an important role.

A shared response in non-immunological genes was observed for parasitic (helminth) and protein sensitization (allergic asthma) models. Considering that both induce a Th2 response, this is not surprising. However, subset D, which shows the strongest response upon these exposures, did not include any Th2-associated cytokines, suggesting that the major expression changes take place downstream of Th2 cytokine signaling. The shared response between helminth infection and allergic asthma involves increased expression of cell cycle-related genes. In these models there is apparently a more rapid turnover or proliferation of cells than in other models. This can be explained by an increased lung epithelial renewal or proliferation, or alternatively by an increase in proliferation for immune cells involved. Although the latter possibility is feasible, the former matches known clinical pathology for asthma models. Asthma is associated with hyperplasia of the mucin-secreting goblet cells [25] and in the studies used this is also described for asthma as well as helminth infection [14], [15]. This is in agreement with the finding that these genes are not found to be induced in *in vitro* models using isolated immune cells.

As pulmonary inflammation often involves leukocyte infiltration, it raises the question whether the common responses occur primarily in sessile lung cells or can be attributed to infiltrating immune cells. Although the common upregulated cluster contains some markers associated with monocytes and macrophages (*e.g. CD14*, *CD68*) or lymphocytes (*e.g. CD72*, *CD80*), these do not show a parallel expression pattern, as would be expected if the gene expression responses are caused by cellular influx. In addition, the common cluster does not include several other markers associated with these types of immune cells, nor those associated with granulocyte lineages, even though several of these are present in the initial 4551 gene set used (*e.g. CD36*, *CD19*, *CD3E*, *CD7*, *CD4*, *CD8A*, *MPO*). For these markers that are not part of the common cluster, the expression changes to the controls are much weaker than those in the common cluster and the responses are also much less consistent across the various models (data not shown). Finally, two of the studies include time points where gene expression responses are at their maximum before a detectable cellular influx is found by pathological analysis, namely the response one day after RSV infection [5] and the early response (2–6 h) after PM exposure [11]. Therefore, it can be concluded that the common infection response can be predominantly attributed to gene expression changes in sessile pulmonary cells rather than to leukocyte influx.

Besides a common up-regulated cluster, we also identified a cluster characterized by a common down-regulation in response to inflammation. This cluster contained a large number of genes involved in growth and development, both of which are important processes for continuous renewal of lung epithelium. As can be seen from Fig. 1, the extent of the down-regulation in this cluster is moderate compared to the effects in the common up-regulated subsets. This suggests that the effects found are not a direct targeted response, but rather a secondary effect that reflects tissue damage or that the induction of an inflammatory response goes at the expense of normally active processes in lung tissue. The finding that the degree of down-regulation is strongest in groups with a stronger up-regulation for subsets A–D and especially E corroborates this assumption.

In comparison with the transcriptional changes found in mice, the effects in primates are generally weaker. More specifically, the inflammatory response-related subset C, which shows the strongest response in mice, shows considerably less up-regulation in macaques. The degree of induction for the interferon signaling-related subset A is also reduced, albeit to a lesser extent. The other subsets show a more moderate response to influenza in both mice and macaques and for these subsets the difference in response is comparatively small, although it is interesting to note that the induction of subset E is virtually absent in macaques. A minor difference in effect is also found for subset Z, which is down-regulated in mice as well as macaques. These differences in expression response can not be ascribed to mere study differences as the effect was reproducible in two studies carried out with different species, namely cynomolgus macaques (*Macaca fascicularis*) in the study by Kobasa *et al.* [10] and pigtailed macaques (*Macaca nemestrina*) in the Baskin *et al.* [8] study. It can not be ascertained whether these differences are characteristic for either influenza or macaques, as microarray data for primate infections with other pathogens were not available. However, it is likely that differences in expression response between mice and macaques can be explained by different disease characteristics between these species. First, differences in effect are most distinct for the two subsets (A and C) where the mouse response upon influenza is strongest, which suggest an association between these responses and disease severity. Second, the influenza studies in mice [9], [16] both reported more severe lung inflammation and pathology than those in macaques [8], [10]. In macaques, the most severe pathology was observed in those infected with the 1918 virus strain. Of the three influenza strains used in the macaque studies, this particular strain causes a gene expression response that compares best to the common mouse response. This corroborates our assumption that the different expression response between rodents and macaques reflects the extent in which lung tissue is infected and/or the virus multiplies.

In conclusion, our study shows that there is a shared *in vivo* expression response to different inducers of lung inflammation. This response comprises several processes involved in host defense and inflammation, and the extent of the response represents the degree of lung inflammation. Our meta-analysis shows considerable overlap with findings from *in vitro* studies (Supporting Dataset S1), especially in cytokines, chemokines, and interferon-induced genes. Some of the differences can be attributed to complex cell-cell interactions, that are absent from *in vitro* systems, such as the induction of the cell division-related subset D. However, as additional microarray data will allow for a more powerful meta-analysis that reveals more common genes for both the *in vivo* and *in vitro* response, genes that do not overlap between the common *in vivo* and *in vitro* response will not always be specific for either response and merely represent the developing knowledge in this field. In the future, additional microarray data from rodents, primates, and perhaps other mammals will contribute to a further understanding of the common *in vivo* response and, ultimately, identification of disease mechanisms that are unique to specific agents or pathogens.

## Methods

We searched Pubmed, GEO (www.ncbi.nlm.nih.gov/geo/), and ArrayExpress (www.ebi.ac.uk/arrayexpress/) for gene expression profiling studies related to acute lung inflammation. If corresponding microarray data were available, they were downloaded from websites indicated by the authors. Data were included in the meta-analysis if they met the following conditions: (a) complete microarray raw or normalized data are available; (b) a suitable uninfected or mock infection control is included in the study; (c) time points are at most eight days after infection (to exclude chronic effects). Furthermore, we excluded experiments with transgenic pathogens or hosts focused on specific research questions, as these typically show inflated responses that are not representative of normal disease. Based on these criteria, we included 45 treatment conditions from 12 experiments [5]–[17]. Of these studies, 4 were carried out in our laboratory and 8 were from the literature. Note that we count the data from the two related articles by Banus *et al.* as one study. Full details of the studies are given in Table 1.

Affymetrix probe sets identifiers were converted to gene symbols using probe set annotation data downloaded from the Affymetrix website (www.affymetrix.com). When necessary, gene symbols in two-color or Affymetrix data files were adjusted to remove tags such as “predicted” and converted to uppercase symbols for further handling. All further calculations were carried out in R [26] or Microsoft Excel. To minimize the influence of data handling procedures, we normalized all raw two-color data with the same algorithm [5], [27]. This consisted of a four-step approach of (1) natural log transformation, (2) quantile normalization of all scans, (3) taking the sample/reference ln-ratio, and (4) averaging replicate spot data. To remove negative values and inflated ratios, MAS4 normalized Affymetrix data were cut off at a lower value of 100, based on the findings of Grundschober *et al.* [28]. MAS5 normalized Affymetrix data were used without adjustment. Affymetrix data were ln-transformed and values for replicate gene symbols were averaged. Finally, for all data sets, the average ln-ratio for treatment to control was calculated per gene. Treatment ratio data for the various studies were merged into one table. To minimize the impact of missing data and non-regulated genes on further analysis, we restricted the initial table of 39312 genes to 4551 genes that were measured in at least 6 out of 12 studies, 30 out of 45 treatment conditions, and had a ratio exceeding ±1.5 in at least one condition. Hierarchical clustering on these 4551 genes was performed in GeneMaths (Applied Maths, St-Martens-Latem, Belgium), using Euclidian distance and Ward linkage. The GeneMaths option Cluster Plot was used to plot the logarithm of the cluster size versus the cluster similarity. This method typically results in a graph with data points that lie closely along a curve that drops off sharply at a level where the number of branches is sufficient to show enough detail, but increasing the number of branches does not result in much additional information. This level can be considered as a recommended cluster similarity cutoff. The resulting value of 88% cluster similarity was used to abridge dendrogram branches above this similarity value. The resulting dendrogram was used to identify branches or clusters with up- or down-regulation that cover multiple studies or pathogens and therefore indicate a common response.

Functional annotation and Gene Ontology (GO) term enrichment analysis were performed with the DAVID website (http://david.abcc.ncifcrf.gov/) [18], [19]. For GO-term enrichment, the functional annotation clustering option was used, with default settings. Functional annotation terms were considered enriched for an Enrichment Score larger than 3, which corresponds to a geometric average p-value of 0.001. MetaCore (GeneGo, San Diego, CA) was used for additional pathway enrichment and visualization.

## Supporting Information

Dataset S1 Genes involved in the common up-regulated infection response
(0.25 MB DOC)

Dataset S2 Genes involved in the common down-regulated infection response
(0.12 MB XLS)

Table S1 Full information on the studies and treatments included
(0.00 MB PDF)
