# Supplementary material for: Identification of a Common Gene Expression Response in Different Lung Inflammatory Diseases in Rodents and Macaques
Source: PLoS One. 2008 Jul 9;3(7):e2596. doi: 10.1371/journal.pone.0002596 (PMC2442866; doi:10.1371/journal.pone.0002596)
Supplement: Table S1 — Full information on the studies and treatments included (0.00 MB PDF) [file pone.0002596.s003.pdf]

Source data	PubMed ID	Species	Exposure	Time point	Order	Letters in Figure 1
Chemical						
Kooter, 2007	17095637	mouse	ozone	12 h	1	A: 12 h
Kooter, 2005	15764483	rat	Particulate matter	2–6 h, 15–21 h, 24–40 h	2-4	B: 2–6 h, C: 15–21 h, D: 24–40 h
Bacterial						
Banus, 2007	17935610 and 17487483	mouse (two strains: C3H and HcB28)	Bordetella pertussis	1 d, 3 d, 5 d	5-10	A: C3H 1 d, B: C3H 3 d, C: C3H 5 d, D: HcB28 1 d, E: HcB28 3 d, F: HcB28 5 d
Lewis, 2008	18029791	mouse	LPS aerosol<br>Mycoplasma pulmonis<br>Pseudomonas aeruginosa	4 h<br>7 d<br>2 h	11<br>12<br>13	G: 4 h<br>H: 7 d<br>I: 2 h
Rosseau, 2007	17163962	mouse	Streptococcus pneumoniae	1 d, 2 d, 4 d	14-16	J: 1 d, K: 2 d, L: 4 d
Viral						
Janssen, 2007	17376894	mouse	Respiratory Syncytial Virus	1 d, 3 d	17,18	A: 1 d, B: 3 d
Kash, 2004	15308742	mouse	Influenza (three strains: 1918, NC, and WSN)	1 d, 3 d	19-24	C: 1918 1 d, D: 1918 3 d, E: NC 1 d, F: NC 3 d, G: WSN 1 d, H: WSN 3 d
Rosseau, 2007	17163962	mouse	Influenza	1 d, 2 d, 4 d	25-27	I: 1 d, J: 2 d, K: 4 d
Baskin, 2004	15367608	macaque	Influenza	4 d, 7 d	38-39	A: 4 d, B: 7 d
Kobasa, 2007	17230189	macaque	Influenza (two strains: K173 and 1918)	3 d, 6 d, 8 d	40-45	C: K173 3 d, D: K173 6 d, E: K173 8 d, F: 1918 3 d, G: 1918 6 d, H: 1918 8 d
Parasitic						
Lewis, 2008	18029791	mouse	Nippostrongylus brasiliensis	5d	28	A: 5 d
Reece, 2006	16926388	mouse	Nippostrongylus brasiliensis	2 d, 3 d, 4 d, 8 d	29-32	B: 2 d, C: 3 d, D: 4 d, E: 8 d
Allergic asthma						
Kuperman, 2005	16083784	mouse	OVA	1 d	33	A: 1 d
Lewis, 2008	18029791	mouse	OVA<br>Aspergillus extract	1 d<br>4 h	34<br>35	B: 1 d<br>C: 4 h
Zimmermann, 2003	12813022	mouse	OVA<br>Aspergillus extract	18 h<br>18 h	36<br>37	D: 18 h<br>E: 18 h
